# Assessment of the Predictive Ability of the Neutrophil-to-Lymphocyte Ratio in Patients with In-Stent Restenosis after COVID-19

**DOI:** 10.3390/diagnostics14202262

**Published:** 2024-10-11

**Authors:** Lyudmila Pivina, Gulnara Batenova, Diana Ygiyeva, Andrey Orekhov, Maksim Pivin, Altay Dyussupov

**Affiliations:** 1 Department of Emergency Medicine, Semey Medical University, Semey 071400, Kazakhstan; gulnara_batenova@mail.ru (G.B.); diana-dikosha@list.ru (D.Y.); pivin97@mail.ru (M.P.); 2 Department of Internal Medicine, Semey Medical University, Semey 071400, Kazakhstan; orekhov-andrei@list.ru; 3 Rector Office, Semey Medical University, Semey 071400, Kazakhstan; altaidusupov@gmail.com

**Keywords:** restenosis, in-stent thrombosis, stenting, coronary artery disease, COVID-19, neutrophil-to-lymphocyte ratio

## Abstract

Background: The neutrophil-to-lymphocyte ratio (NLR) is an independent predictor of the severity of coronary heart disease and COVID-19. This study aims to assess the predictive ability of the NLR in patients with in-stent restenosis after COVID-19. Materials and Methods: a cross-sectional study included 931 patients who underwent repeated myocardial revascularization between May 2020 and May 2023. The 420 patients of the main group had in-stent restenosis, of which 162 patients had COVID-19 previously. The control group included 511 patients without stent restenosis (107 patients had COVID-19 previously). All reported events were verified by hospital electronic records from the Complex Medical Information System. Results: The mean values of the NLR were 2.51 and 2.68 in the study groups, respectively. A statistically significant positive relationship in both groups was found between the NLR and troponin, D-dimer, C-reactive protein, creatinine, ALT, and AST. A statistically significant positive relationship was found between NLR and myocardial infarction (MI) in patients of both groups (*p* = 0.004; *p* < 0.001, respectively) and a negative relationship with the ejection fraction (*p* = 0.001; *p* < 0.036, respectively). An evaluation of the predictive ability of the clinical and laboratory predictors of recurrent myocardial infarction shows a high degree of utility of this model. The area under the ROC curve for AUC for NLR was 0.664 with 95% CI from 0.627 to 0.700 (*p* < 0.001). Conclusions: NLR is one of the significant factors for predicting the development of adverse outcomes in patients with revascularized myocardium after COVID-19.

## 1. Introduction

Coronary artery disease (CAD) is characterized by the reduction of blood flow to the heart muscle due to atherosclerotic plaque in the arteries of the heart or a spasm of coronary arteries. The problem of high morbidity and mortality from CAD is still one of the most important for health systems worldwide, which is associated with a large number of risk factors for the population, including an increase in the proportion of elderly and senile people, social factors, and stress [1]. One of the most effective methods for treating CAD is percutaneous coronary interventions to restore blood flow in the arteries [2]. The use of stents coated with drugs that prevent thrombus formation has increased the effectiveness of this method: the rates of stent thrombosis, regardless of the timing of their development, range from 1 to 4%, and the risk of mortality increases significantly with multivessel lesions [3]. However, even with dual or triple antiplatelet therapy, there is an increase in the prevalence of stent restenosis, which is a narrowing of the lumen of the coronary vessel by more than 50% according to an angiographic examination [4]. Within five years after stenting, the frequency of recurrence of stenosis and thrombosis of the coronary arteries has reached 15–20%, while the mortality rate is about 45% [5,6,7,8].

One of the most important mechanisms of coronary artery stent restenosis is the vascular intima’s inflammatory process associated with coagulation system activation. The release of inflammatory mediators, migration of neutrophils, and monocytes, and the accumulation of platelets, lymphocytes, and macrophages [9] accompany microdamages to the vascular wall during stenting. Cytokine secretion also enhances smooth muscle cell migration from the media to the intima and their accumulation and proliferation. Given that complete endothelialization of drug-eluting stents can take up to two years, the risks of restenting remain high for an extended period [10].

The COVID-19 pandemic is an epidemic that has affected the vast majority of the world, caused by the spread of the SARS-CoV-2 coronavirus in 2020–2022. The outbreak of the virus was first recorded in Wuhan, China, in December 2019 [11]. The coronavirus pandemic has contributed negatively to in-stent restenosis and thrombosis rates worldwide [12]. During the pandemic, myocardial revascularization decreased due to a shortage of healthcare resources and patient refusal to be hospitalized for fear of infection [13]. However, the rate of stent thrombosis during this period increased to 21%, but the studies were conducted on small and heterogeneous samples [14]; this problem requires more comprehensive research.

Some studies have shown that the neutrophil-to-lymphocyte ratio (NLR), calculated as a simple ratio of the absolute number of these parameters in peripheral blood, is an independent predictor of the severity of coronary heart disease, the total occlusion of coronary vessels with the development of myocardial infarction (MI) [15,16,17,18,19].

The NLR was an independent risk factor for the severe current of COVID-19 [20]. In most COVID-19 patients, the NLR was greater than 2.97, which was associated with the disease severity [21]. The results of a systematic review with a meta-analysis showed that this indicator was an independent predictor of mortality in COVID-19 [22]. AN increased NLR in COVID-19 may be due to the direct infection of the bone marrow with abnormal hematopoiesis or an autoimmune reaction against lymphocytes [23], or the dysregulation of the cytokine expression associated with lymphocyte death [24]. Moreover, the NLR was identified in a meta-analysis as a prognostic biomarker for patients with sepsis [25].

Our study aims to assess the predictive ability of the NLR in patients with in-stent restenosis after COVID-19.

## 2. Materials and Methods

### 2.1. Characteristics of the Study Groups

We conducted a cross-sectional study in a targeted sample of patients with CAD who underwent repeat myocardial revascularization between May 2020 and May 2023. A total of 931 patients were included in the study. The 420 patients included in the main group had coronary artery stent restenosis or thrombosis that led to repeat revascularization, of which 162 patients had a history of COVID-19; 258 did not have this infection. The control group included 511 patients with repeat myocardial revascularization without stent restenosis or thrombosis. Of these, 107 patients had a previous history of COVID-19; 404 patients did not have a history of this infection. At the time of the study, all patients had no clinical or laboratory signs of acute infection and, therefore, none of them were taking corticosteroids or other drugs that could affect the NLR rate.

Exclusion criteria: individuals with autoimmune systemic diseases, cancer patients, and patients who refused to participate in the study.

After risk stratification, all patients underwent coronary angiography (CAG) followed by myocardial revascularization with stenting. A study participant card was created for each patient. The patients provided informed consent and permission to publish. More than 60% of patients were in the age group of 51–70 years, with a more than threefold predominance of males. More than 70% of patients were on old age or disability pensions (Table 1). The age distribution of all patients included in our study belongs to the category of normal distribution. The average age of all patients included in the study was 64.31 ± 8.19 years. For women, this rate was 67.07 ± 10.48 years. For men, the average age was 63.39 ± 9.92 years.

### 2.2. Collection of Clinical and Laboratory Data

The clinical data of patients were collected from an electronic medical database, including demographics, clinical symptoms and signs, comorbidities, imaging findings, laboratory tests, clinical outcomes, and information on previous myocardial revascularization and coronavirus infection. For all patients, the diagnosis was made by experienced specialists. All reported events were verified by hospital electronic records from the Complex Medical Information System and adjudicated by two cardiologists in consensus.

Venous blood samples were collected for all patients in the frame of 10 min after admission. The laboratory tests included a complete blood count, high-sensitivity troponin I, D-dimer, creatine kinase (CK), creatine kinase-MB (CK-MB), serum creatinine and glucose, ESR, C-reactive protein, Alanine aminotransferase (ALT), Aspartate aminotransferase (AST), and fibrinogen. Renal dysfunction was defined as an elevated serum creatinine >1.3 mg/dL for men and >1.1 mg/dL for women. The NLR was defined by the absolute neutrophil count divided by the lymphocytes. LVEF (left ventricular ejection fraction) values were obtained using transthoracic echocardiography performed after hospitalization.

### 2.3. Statistical Analysis

For all continuous variables, the mean and confidence intervals were calculated depending on the type of data distribution. For variables with a distribution deviating from normal, the median and interquartile range were determined. Qualitative variables were analyzed by calculating the absolute and relative indicators. For categorical variables, data were presented as absolute and relative numbers. For qualitative data, the significance of differences in the groups was determined by performing the Chi-square test (χ^2^). For quantitative data, central tendencies were measured. A comparison of laboratory parameters was performed using the nonparametric Mann–Whitney test. A multivariate regression analysis was used to assess the relationship between clinical and laboratory parameters and the NLR. The prognostic value of the NLR was assessed using ROC analysis. Statistical significance was set at *p* < 0.05. All statistical calculations were performed using SPSS version 20.0 software (IBM Ireland Product Distribution Limited, Dublin, Ireland).

### 2.4. Ethical Approval Details

This study was conducted according to the guidelines of the Declaration of Helsinki and approved by the Local Ethics Commission of the Semey Medical University on 16 March 2022. Protocol N 7.

All participants signed a voluntary informed consent form. They were informed about the processing of the data collection, with the subsequent publication of the results of the study without specifying personal data.

## 3. Results

The median values of the NLR did not have statistically significant differences in the main and control study groups (Table 2).

The results of the linear regression analysis of the relationship between the NLR and laboratory tests are presented in Table 3. A statistically significant positive relationship in both groups was found for troponin, D-dimer, C-reactive protein, creatinine, ALT, and AST. Concerning CPK, a statistically significant relationship was found only in the control group.

**Figure 1:**
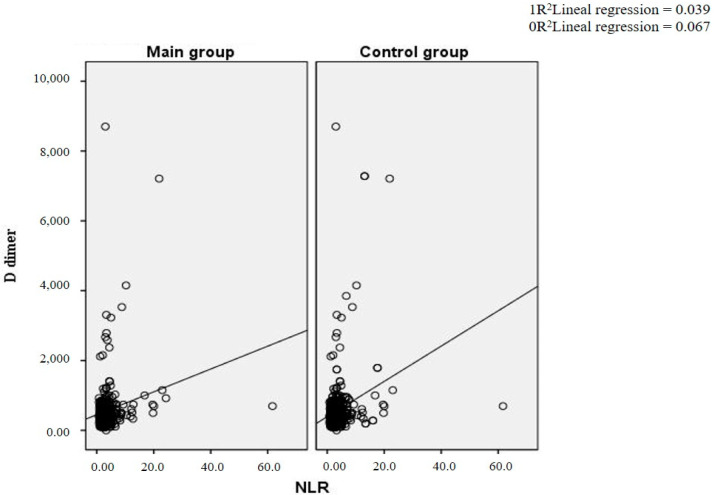
Scattergram of the dependence of D-dimer and NLR.

Figure 1 presents a scatter plot describing the relationship between the NLR and D-dimer in both the main and control groups. It indicates that most data points are concentrated in the lower range of both axes, especially at NLR values less than 10 and D-dimer levels less than 2000. The graphs show a weak positive correlation between the NLR and D-dimer levels in both groups. The coefficient of determination (R²) in the study group is 0.039, which means that the NLR explains 3.9% of the variability in D-dimer levels, indicating a weak linear relationship. In the control group, the coefficient of determination (R²) is 0.067, which means that the NLR explains 6.7% of the variability in D-dimer levels, indicating a weak linear relationship.

**Figure 2:**
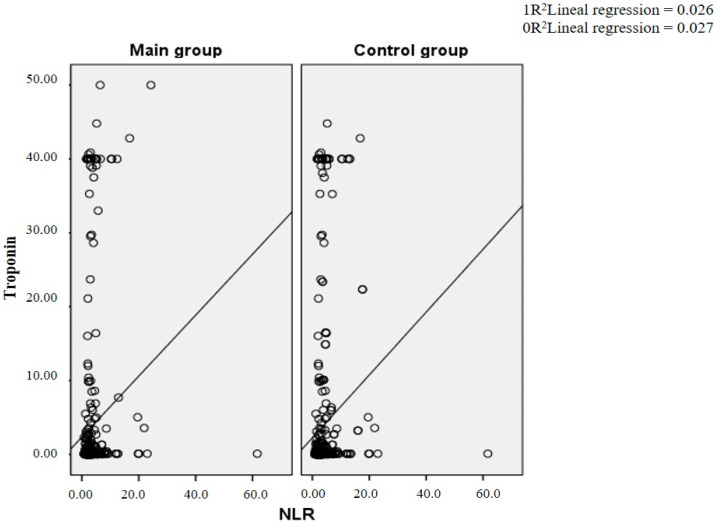
Scattergram of the dependence of troponin and NLR.

Figure 2 shows the relationship between the NLR and the troponin level. The values are highly scattered in both the main and control groups, indicating the instability of this relationship despite its statistical significance. Most of the data points are concentrated in the lower range of both axes, especially for NLR values less than 10 and troponin I levels less than 10. Several outliers are outside these limits, especially for high NLR and troponin I levels. In both groups, the plot indicates a weak positive correlation between the NLR and troponin I levels. The coefficient of determination (R²) in the study group was 0.026, meaning that the NLR explained 2.6% of the variability in troponin I levels, indicating a weak linear relationship. In the control group, this coefficient was 0.027, meaning that the NLR explained 2.7% of the variability in troponin I levels, indicating a weak linear relationship.

**Figure 3:**
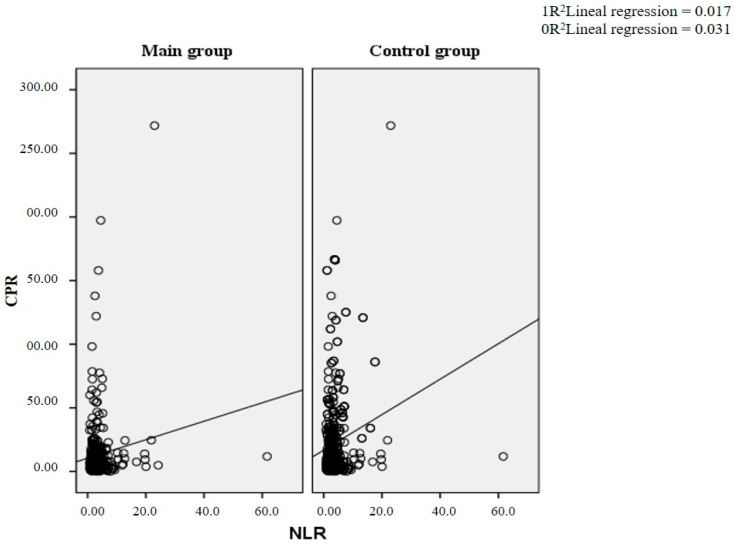
Scattergram of the dependence of CPR and NLR.

The relationship between the NLR and the C-reactive protein in both the main and control groups also showed a significant spread of indicators. The slope of the approximation line was lower than in the case of determining the relationship with troponin (Figure 3). The graph shows a linear regression line indicating a weak positive correlation between the NLR and CRP levels. The coefficient of determination (R²) is 0.017, meaning that the NLR explains 1.7% of the variability in CRP levels, indicating a weak linear relationship. In the control group, the coefficient of determination (R²) is 0.031, meaning that the NLR explains 3.1% of the variability in CRP levels, indicating a weak linear relationship.

The linear regression analysis between the NLR and clinical parameters demonstrates the presence of a statistically significant positive relationship between the parameter and the development of MI in patients of both the main group (*p* = 0.004) and the control group (*p*-*p* < 0.001) and a negative relationship with the ejection fraction in both groups (*p* = 0.001; *p* < 0.001, respectively) (Table 4). The regression coefficient for myocardial infarction was 1.183 and 1.386, respectively.

The results of the multiple regression analysis of the relationship between MI and the demographic, clinical, and laboratory parameters indicate a statistically significant contribution of increased levels of the NLR, glucose, creatinine, C-reactive protein, and LDL, previous COVID-19, and decreased left ventricular EF in the development of this unfavorable outcome in people with previous revascularization myocardium (Table 5).

**Figure 4:**
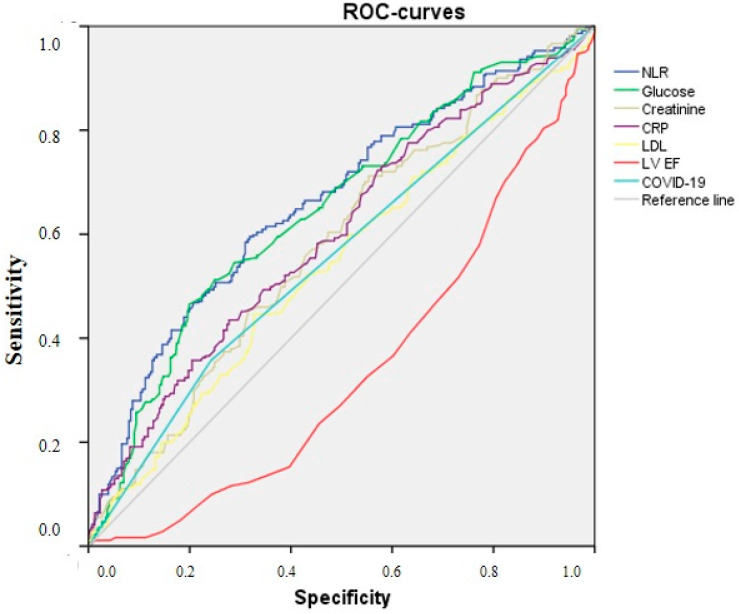
ROC curve and AUC for the model of myocardial infarction development depending on the laboratorial and clinical indicators.

An analysis of the model for the development of MI depending on the laboratorial and clinical indicators showed their good prognostic ability. The area under the ROC curve for AUC for the NLR was 0.664 with 95% CI from 0.627 to 0.700 (*p* < 0.001), indicating that the NLR may be a useful predictor for future recurrent MI. For the glucose level, the area under the ROC curve for AUC was 0.649, CI [0.612; 0.685]; for creatinine it was 0.581, CI [0.543; 0.618]; for CRP it was 0.600, CI [0.562; 0.637]; for LDL it was 0.542, CI [0.504; 0.581]; and for previous COVID-19 it was 0.558, CI [0.519; 0.596]. A left ventricular ejection fraction was inversely associated with the development of MI. The area under the ROC curve for this indicator was 0.343, CI [0.308; 0.378] (Figure 4).

## 4. Discussion

The NLR is a biomarker characterizing the state of both the innate immune response supported by neutrophils and the adaptive immunity mediated by lymphocytes [26]. Neutrophils are the main regulatory and effector cells responsible for the immune system’s response to systemic inflammation through various mechanisms such as cytokine secretion, phagocytosis, and chemotaxis [27]. The activation of immune cells by neutrophils enhances the functions of mesenchymal stem cells, B cells, killer cells, CD4, and CD8 [28].

An increase in the number of neutrophils leads to an increase in the NLR index in such pathological conditions as, first of all, bacterial infection, cerebrovascular accidents, myocardial infarction, multiple injuries, and oncological diseases accompanied by the activation of the systemic inflammatory response [29,30,31].

An increase in the NLR is usually accompanied by an increase in the number of neutrophils and a decrease in lymphocytes. According to some studies, the NLR can be associated with mortality from cardiovascular diseases (HR 1.1; 95% CI 1.06–1.29 per NLR quartile), chronic respiratory diseases (1.24; 1.04–1.47), and genitourinary diseases (1.62; 1.21–2.17) [30]. Moreover, a significant association of this indicator with an increase in the overall mortality has been found (HR 1.64 per NLR quartile; 95% CI 1.44–1.86) [32]. On the other hand, no significant association of the NLR with mortality from cancer, central nervous system diseases, or diabetes mellitus has been found [30]. The NLR may serve as an indicator of acute physiological stress even earlier (less than 6 h) than the C-reactive protein [33]. A decrease in the NLR over time is usually associated with a favorable prognosis, reflecting the restoration of immune balance.

Normal threshold values of the NLR are still under discussion. Their range in adults is from 0.78 to 3.55 [34], while the average NLR value in the general population is 1.76 [0.83; 3.92]. In men, this indicator is usually slightly higher than in women (1.88 vs. 1.68) [35]. In our study, in individuals with coronary artery restenosis and with repeat revascularization without restenosis, the median values were 2.51 and 2.68, respectively, which is significantly higher than the average population values; the differences in the groups were not statistically significant, which can be explained by the presence of individuals who had undergone the coronavirus infection in both study groups. Factors such as the age of patients, the use of corticosteroids, hematological diseases, chemotherapy, and HIV infection may influence the increase in the rate [36]. The ages of the patients included in our study were approximately the same; none of them had a history of cancer or HIV infection. None of the study participants had acute forms of COVID-19 or were taking corticosteroids or other drugs at the time of enrollment that could have affected the NLR value.

It is known that COVID-19-associated pneumonia is characterized by a decrease in the number of lymphocytes; this indicator has a negative correlation with the severity of the disease, which gives reason to consider it as a predictor of the severity of COVID-19 [37]. A decrease in the number of T and NK cells is observed to a greater extent compared to B lymphocytes, which indicates a loss of control over the viral infection [37,38]. At the same time, high levels of circulating inflammatory cytokines, procalcitonin, CRP, and serum ferritin contributed to a decrease in defense mechanisms and the accumulation of neutrophils, which ultimately led to an increase in the values of the NLR in patients with COVID-19-associated pneumonia [39,40].

A clinical study conducted by Italian researchers demonstrated that patients with COVID-19-associated pneumonia had a significant decrease in neutrophils, leukocytes, and lymphocytes compared to patients with community-acquired pneumonia. However, bacterial infection always leads to an increase in the number of neutrophils and leukocytes, and accordingly, to an increase in the NLR; so, this indicator can be considered a very simple and rapid tool for the differential diagnosis of viral and bacterial pneumonia [41].

In addition to infectious and inflammatory diseases, cardiovascular events have also been associated with an increase in the NLR. Thus, it was found that this indicator, exceeding 4.5, can be an independent predictor of death due to coronary heart disease for a long period (up to 14 years) [42]. It has been shown that the NLR can help to more accurately classify patients from the intermediate risk category according to the Framingham risk score, indicating the probability of cardiovascular mortality within 10 years, regardless of the previous revascularization [42,43,44]. In addition, the prognostic role of NLR has also been assessed in patients with diabetes mellitus concerning adverse cardiovascular events [43].

An increase in the NLR at hospital admission above 2.97 (AUC = 0.714. *p* = 0.001) was found to be an independent predictor of 3-month all-cause mortality in patients with arterial hypertension over 80 years of age [45].

The main mechanism of the association of the NLR with the cardiovascular disease outcomes is inflammation and associated oxidative stress, leading to endothelial dysfunction. The activation of the NPL3 inflammasome (a multiprotein oligomeric complex responsible for the activation of the inflammatory response) leads to the increased synthesis and release of the interleukin family, in particular, IL-1, and an imbalance between the interleukins and their antagonists [46].

The results of our analysis demonstrate a statistically significant positive relationship between the NLR indicator and the level of troponin, D-dimer, C-reactive protein, creatinine, ALT, and AST both in the group of patients with coronary artery restenosis and in the control group. The relationship between the NLR with troponin, D-dimer, and creatinine in individuals of both groups showed a high degree of homogeneity of the results, which indicates the stability of such a relationship. The same results were established regarding the relationship between the NLR, AST, and ALT, which indicates the involvement of the liver function in the inflammatory process in patients with coronary artery revascularization against the background of previous COVID-19, even in the late period.

The results of our multiple regression analysis demonstrated that the NLR is not the only predictor of adverse cardiovascular events in our study participants. Such parameters as previous a COVID-19 infection, low-density lipoproteins, CRP, low ejection fraction, and elevated glucose and creatinine levels also contributed to their development. Therefore, we can consider the NLR as one of the main predictors of myocardial infarction in patients with previously revascularized myocardium. This indicator could be recognized as a prognostic marker of both COVID-19 and adverse cardiovascular events, which is supported by the results of our study and by data from other authors [47,48]. The calculation of this indicator is the most accessible compared to other tests, which makes it useful in emergencies.

## 5. Conclusions

The results of our analysis demonstrate a statistically significant positive relationship between the NLR and the level of troponin, D-dimer, C-reactive protein, creatinine, ALT, and AST in both study groups. A statistically significant relationship was found for the NLR with such clinical indicators as myocardial infarction in the main group and an ejection fraction in both study groups, but more pronounced in the main group.

An evaluation of the predictive ability of the clinical and laboratory predictors of recurrent myocardial infarction shows a high degree of utility of this model based on a ROC curve analysis. The NLR is one of the significant factors for predicting the development of adverse outcomes in patients with revascularized myocardium after COVID-19. However, assessing the relationship of this indicator with the possibility of restenosis or stent thrombosis after COVID-19 requires larger-scale studies in the future.

## Figures and Tables

**Table 1:** Socio-demographic characteristics of patients included in the study (*N* = 931).

Rates		*N*	%
Age (years)	<50	76	8.17
	51–70	592	63.58
	71>	263	28.25
Sex	male	700	75.18
	female	231	24.82
Job status	disabled person	76	8.17
	pensioner	508	54.56
	unemployed	133	14.28
	working	214	22.99

**Table 2:** Characteristics of the NLR in the study groups.

Rate	Main Group		Control Group		*p*
	Median	Interquartile Range	Median	Interquartile Range	
NLR	2.51	1.6	2.68	1.8	0.166

**Table 3:** Results of linear regression analysis between NLR and laboratory parameters.

Rate	r Squared		B		*p*	
	Studied Groups					
	Main	Control	Main	Control	Main	Control
Troponin	0.026	0.027	0.062	0.062	0.001	0.001
D-dimer	0.039	0.067	0.001	0.001	0.001	0.001
CPK	0.01	0.019	0.001	0.001	0.203	0.002
CPK-MB	0.007	0.006	0.005	0.003	0.28	0.09
Platelets	0.008	0.0013	0.001	0.001	0.917	0.773
IgG	0.014	0.001	0.0011	0.001	0.892	0.626
IgM	0.06	0.003	−0.118	−0.304	0.455	0.302
CRP	0.017	0.031	0.024	0.022	0.007	0.001
Fibrinogen	0.013	0.001	−0.001	0.01	0.872	0.965
APTT	0.06	0.001	0.013	0.007	0.343	0.56
Creatinine	0.087	0.022	0.011	0.009	0.001	0.001
ALT	0.05	0.063	0.031	0.028	0.001	0.001
AST	0.051	0.017	0.014	0.008	0.001	0.004

CPK—creatinine phosphokinase; CPK-MB—creatine phosphokinase-MB; CRP—C-reactive protein; APTT—activated Partial Prothrombin Time; ALT—Alanine aminotransferase; AST—aspartate aminotransferase.

**Table 4:** Regression analysis of the relationship between NLR and clinical indicators.

Rate	B		Confidential Intervals				*p*	
	Studied Groups							
	Main	Control	Main		Control		Main	Control
			Lower Limit	Upper Limit	Lower Limit	Upper Limit		
Mortality	−0.716	−0.414	−1.683	0.25	−1.464	0.636	0.146	0.439
MI	1.183	1.386	0.375	1.991	0.696	2.075	0.004	0.000
Number of stents	0.054	−0.08	−0.445	0.553	−0.531	0.363	0.832	0.712
Ejection Fraction	−0.071	−0.072	−0.112	−0.03	−0.108	−0.036	0.001	0.000
Previous CVI	0.02	0.018	0.972	1.070	0.970	1.068	0.418	0.462

**Table 5:** Multivariate regression analysis of the association between MI and demographic, clinical, and laboratorial parameters.

Predictors	Unadjusted		Adjusted	
	COR; 95% CI	*p*	AOR; 95% CI	*p*
Age	1.003;0.990–1.016	0.654	1.005; 0.990–1.021	0.515
Hypertension	0.420; 0.170–1.038	0.060	0.386; 0.143–1.043	0.060
Diabetes mellitus	0.662; 0.479–0.913	0.012 *	0.879; 0.582–1.327	0.539
NLR	1.124; 1.068–1.182	<0.001 *	1.072; 1.014–1.133	0.014 *
Glucose	1.154; 1.099–1.212	<0.001 *	1.102; 1.038–1.171	0.002 *
Urea	1.064; 1.019–1.112	0.005 *	0.941; 0.874–1.013	0.108
Creatinine	1.006; 1.002–1.010	0.001 *	1.005; 1.000–1.009	0.047 *
C-reactive protein	1.015; 1.009–1.021	<0.001 *	1.012; 1.006–1.018	<0.001 *
Triglycerides	1.000; 0.926–1.080	0.996	1.015; 0.929–1.108	0.744
LDL	1.158; 1.009–1.328	0.036 *	1.229; 1.052–1.436	0.009 *
HDL	0.599; 0.401–0.896	0.013 *	0.673; 0.432–1.048	0.080
Previous COVID-19	1.742; 1.305–2.326	<0.001 *	1.645; 1.198–2.257	0.002 *
Men	1.165; 0.855–1.587	0.334	1.146; 0.813–1.618	0.436
LV ejection fraction	0.945; 0.931–0.960	<0.001 *	0.957; 0.941–0.972	<0.001 *

*—the influence of the predictor is statistically significant (*p* < 0.05).

## Data Availability

The data are not publicly available due to confidentiality agreements and privacy concerns but can be accessed upon reasonable request to ensure proper use and adherence to ethical guidelines.
